# Ependymoma associated protein Zfta is expressed in immature ependymal cells but is not essential for ependymal development in mice

**DOI:** 10.1038/s41598-022-05526-y

**Published:** 2022-01-27

**Authors:** Vicente Herranz-Pérez, Jin Nakatani, Masaki Ishii, Toshiaki Katada, Jose Manuel García-Verdugo, Shinya Ohata

**Affiliations:** 1 Laboratory of Comparative Neurobiology, Institute Cavanilles of Biodiversity and Evolutionary Biology, CIBERNED, University of Valencia, 46980 Paterna, Spain; 2 Department of Cell Biology, Functional Biology and Physical Anthropology, University of Valencia, 46100 Burjassot, Spain; 3 Department of Biomedical Sciences, College of Life Sciences, Ritsumeikan University, Shiga, 525-8577 Japan; 4 Molecular Cell Biology Laboratory, Research Institute of Pharmaceutical Sciences, Faculty of Pharmacy, Musashino University, Tokyo, 202-8585 Japan; 5 Department of Physiological Chemistry, Graduate School of Pharmaceutical Sciences, University of Tokyo, Tokyo, 113-0033 Japan

**Keywords:** Development of the nervous system, CNS cancer, CNS cancer

## Abstract

The fusion protein of uncharacterised zinc finger translocation associated (ZFTA) and effector transcription factor of tumorigenic NF-κB signalling, RELA (ZFTA-RELA), is expressed in more than two-thirds of supratentorial ependymoma (ST-EPN-RELA), but ZFTA’s expression profile and functional analysis in multiciliated ependymal (E1) cells have not been examined. Here, we showed the mRNA expression of mouse *Zfta* peaks on embryonic day (E) 17.5 in the wholemount of the lateral walls of the lateral ventricle. Zfta was expressed in the nuclei of FoxJ1-positive immature E1 (pre-E1) cells in E18.5 mouse embryonic brain. Interestingly, the transcription factors promoting ciliogenesis (ciliary TFs) (e.g., multicilin) and ZFTA-RELA upregulated luciferase activity using a 5′ upstream sequence of *ZFTA* in cultured cells. *Zfta*^*tm1/tm1*^ knock-in mice did not show developmental defects or abnormal fertility. In the *Zfta*^*tm1/tm1*^ E1 cells, morphology, gene expression, ciliary beating frequency and ependymal flow were unaffected. These results suggest that Zfta is expressed in pre-E1 cells, possibly under the control of ciliary TFs, but is not essential for ependymal development or flow. This study sheds light on the mechanism of the ZFTA-RELA expression in the pathogenesis of ST-EPN-RELA: Ciliary TFs initiate ZFTA-RELA expression in pre-E1 cells, and ZFTA-RELA enhances its own expression using positive feedback.

## Introduction

Ependymal (E1) cells are multiciliated epithelial cells that line the walls of the brain ventricles and help circulate the cerebrospinal fluid (CSF) through the beating of their motile cilia^1,2^. In the ventricular-subventricular zone (V-SVZ) of the lateral ventricle (LV), E1 cells surround the adult neural stem (B1) cells, forming a pinwheel-like adult neural stem cell niche^3^, and contribute to the promotion of adult neurogenesis and guidance of migrating new neurons^3–6^. E1 cells differentiate from radial glial cells, which are embryonic neural stem cells in the mid-to-late embryonic development^7–9^. Mutations in genes involved in the development of E1 cells can cause severe neurological disorders, such as hydrocephalus and ependymoma^1,10^. The characterisation of these genes is important to understand the development of the V-SVZ and the pathogenesis of these diseases.

*Zinc finger translocation associated* (*ZFTA*, formerly known as *chromosome 11 open reading frame 95*, *C11orf95*) is mutated in chondroid lipoma and ependymoma^11–15^. In chondroid lipoma, a reciprocal translocation, t(11;16)(q13;p13), generates the fusion proteins of ZFTA and myocardin-related transcription factor B (MRTFB, formerly known as MKL2). In more than two-thirds of supratentorial ependymoma, which is classified as ST-EPN-RELA, several forms of fusion proteins of ZFTA and RELA, an effector transcription factor of the inflammatory NF-κB pathway, have been identified. By constitutively localising to the nucleus, ZFTA-RELA causes the hyper-activation of the NF-κB signalling pathway and eventually ST-EPN-RELA^12,16–18^. ZFTA also forms fusion proteins with mastermind-like transcriptional coactivator 2 (MAML2), transcriptional activator MN1, nuclear receptor coactivator 1 and 2 (NCOA1/2) and Yes1 associated transcriptional regulator (YAP1)^12,19–21^. These fusion proteins are involved in the formation of other subtypes of ependymomas. Mouse models of ependymoma have been generated by the transplantation of ZFTA-RELA-expressing neural stem cells into adult mouse brain^12^ and the exogenous expression of ZFTA-RELA in neonatal mouse brain^22,23^. Despite these reports, the expression profile and potential function of *ZFTA* in the development of E1 cells have yet to be elucidated.

Here, we show that mouse *Zfta* is expressed in embryonic immature ependymal (pre-E1) cells, possibly under the control of ciliary transcription factors (TFs). *Zfta*^*tm*1^ knock-in mice are apparently normal in their development and fertilisation. Our study suggests a molecular mechanism by which ZFTA-RELA is expressed in pre-E1 cells in the pathogenesis of ST-EPN-RELA. Inhibition of the ZFTA portion of ZFTA-RELA may be a therapeutic strategy for the brain tumours with poor prognoses.

## Results

### Zfta is expressed in the nucleus of mouse pre-E1 cells

**Figure 1:**
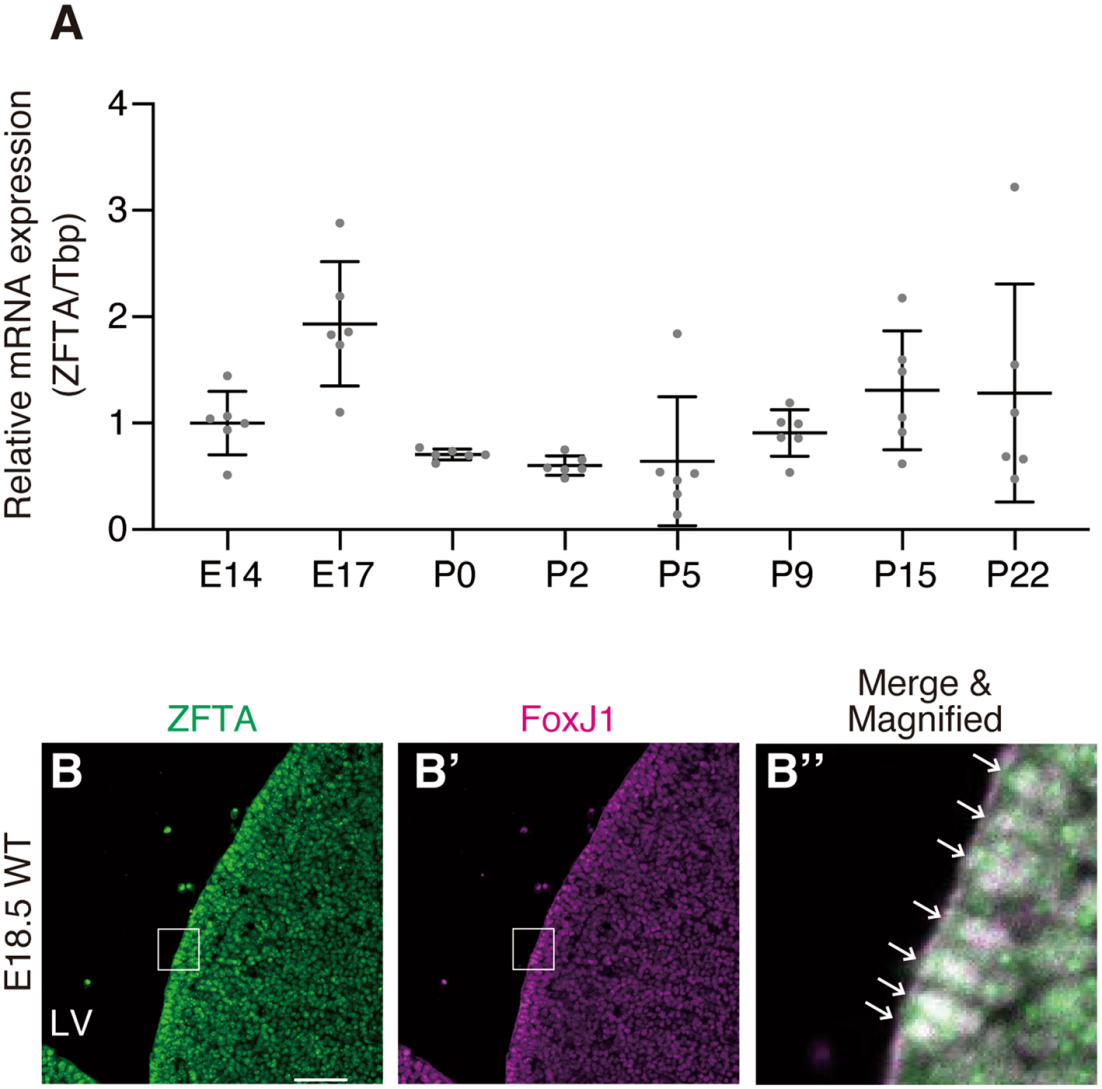
Expression of mouse Zfta in embryonic pre-E1 cells. (**A**) Expression profile of *Zfta* mRNA in the anterior portion of the lateral walls of LV assessed by qPCR. *Tbp* was used as an internal control. Data shown are the mean ± standard deviation (SD). Each point on the graph is the relative *Zfta* expression level of an individual mouse. N = 6 each. (**B**–**B**″) Coronal cryosections of wildtype (WT) E18.5 mouse embryos were stained with anti-human ZFTA (green, **B**) and anti-FoxJ1 (magenta, **B**’) antibodies. The merged and magnified image of the region indicated by white boxes in **B** and **B**’ is shown in **B**″. White arrows in **B**″ indicate the co-expression of ZFTA and FoxJ1 in pre-E1 cells’ nuclei (shown in white). Bar = 100 µm.

In the mouse LV, radial glial cells are committed to E1 cells around embryonic day (E) 15.5^7–9^. These immature E1 cells (pre-E1 cells) express a TF, Forkhead box protein J1 (FoxJ1), retain radial-glial morphology until birth, and morphologically differentiate into mature E1 cells by postnatal day (P) 5^7,24^. To address the expression profile of mouse *Zfta* mRNA in the LV walls, we performed a quantitative polymerase chain reaction (qPCR) using cDNAs synthesised from the anterior part of the LV walls on E14.5, E17.5, P0, P2, P5, P9 and P15 (Fig. 1A). Interestingly, the relative expression level of *Zfta* mRNA peaked on E17.5, which was between the fate determination (around E15.5) and the morphological differentiation (after P0) of pre-E1 cells. Through immunohistochemistry, we examined the expression of Zfta in the E18.5 embryonic brain using the anti-human C11orf95/ZFTA antibody, which specifically recognises both human ZFTA and mouse Zfta in immunocytochemistry and immunoblotting (Fig. S1), and found that FoxJ1-positive cells lining the LV walls expressed Zfta (Fig. 1B–B″). These results suggest that Zfta is expressed in mouse pre-E1 cells.

### Multiciliate differentiation and DNA synthesis associated cell cycle protein (MCIDAS)-responsive elements upstream of human ***ZFTA***

**Figure 2:**
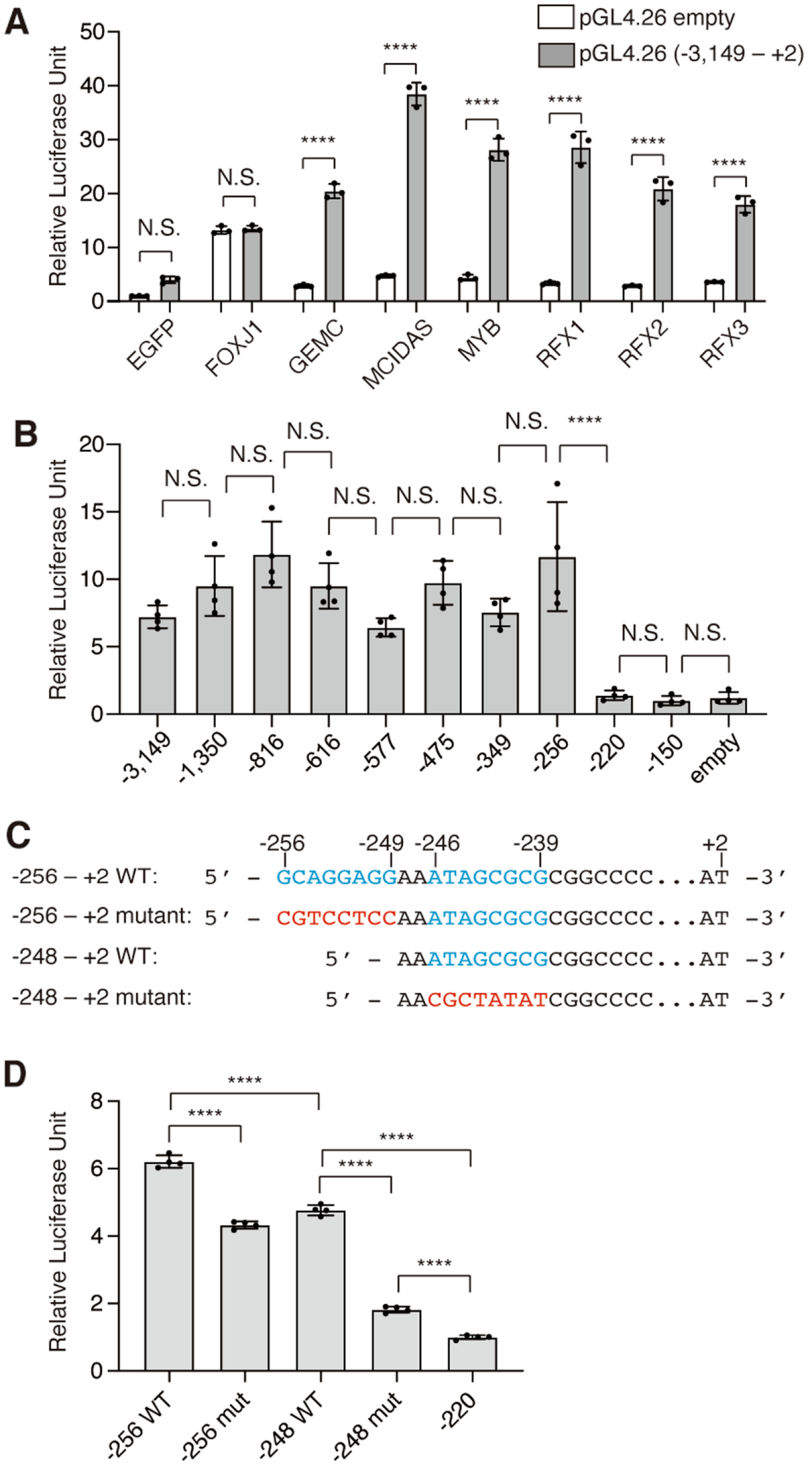
Transcriptional regulation of *ZFTA* by ciliary transcription factors. (**A**) Transactivation of pGL4.26 *ZFTA* (− 3149 to +2) by ciliary TFs in HeLa cells. Plasmid encoding EGFP or indicated ciliary TFs were co-transfected with pGL4.26 or pGL4.26 *ZFTA* (− 3149 to +2) to HeLa cells. The signal of each sample was normalised by the mean of the signals obtained from cells co-transfected with pGL4.26 and EGFP. Data shown are the mean ± SD. Each point on the graph is the relative luciferase activity of an individual sample. N = 3 each. ****p < 0.0001; N.S., not significant. (**B**) Identification of MCIDAS-responsive region upstream of ZFTA. Plasmid encoding MCIDAS was co-transfected with a series of deletion mutants for pGL4.26 *ZFTA* (− 3149 to +2) to HeLa cells. Luciferase activities were normalized by the mean of the activity obtained in HeLa cells co-transfected with pGL4.26 and MCIDAS. The labels on the graphs indicate only the start of the ZFTA upstream sequence. For example, pGL4.26 ZFTA (− 3149 to +2) is labeled as “− 3149”. Data shown are the mean ± SD. Each point on the graph is the relative luciferase activity of an individual sample. N = 4 each. ****p < 0.0001; N.S., not significant. (**C**) Schematic diagram of the constructs used in (**D**). Cyan and red letters indicate WT and mutant sequences, respectively. (D) MCIDAS-responsive luciferase assay using the constructs in (**C**). Luciferase activities were normalized by the mean of the activity obtained in HeLa cells co-transfected with pGL4.26 *ZFTA* (− 220 to + 2) and MCIDAS. Data shown are the mean ± SD. Each point on the graph is the relative luciferase activity of an individual sample. N = 4 each. ****p < 0.0001.

Several TFs have been shown to promote the differentiation of multiciliated cells, including E1 cells, called ciliary TFs^25,26^. For example, Multicilin, which is encoded by *MCIDAS*, and Myb are expressed in FoxJ1-positive pre-E1 cells in embryos and immature E1 cells in neonatal mice, respectively, and promote the differentiation of E1 cells^27,28^. To examine whether ciliary TFs can promote the expression of *ZFTA*, we cloned an upstream sequence of human *ZFTA*. As the transcription start point of *ZFTA* has not been experimentally determined, in the present study, we defined the nucleotide immediately upstream of the start codon of *ZFTA* as ‘− 1’ and the initial nucleotide of the start codon as ‘+1’. We cloned the *ZFTA* upstream sequence from − 3149 to +2 into the pGL4.26 luciferase reporter plasmid [hereafter referred to as pGL4.26 *ZFTA* (− 3149 to +2)], co-transfected it with plasmids encoding enhanced green fluorescent protein (EGFP), FOXJ1, geminin coiled-coil domain containing (GMNC, formerly known as GemC1), MCIDAS, MYB, regulatory factor X1 (RFX1), RFX2, and RFX3, and performed luciferase assay. In cells expressing GMNC, MCIDAS, MYB, RFX1, RFX2, and RFX3, but not EGFP or FOXJ1, luciferase activity was significantly higher in samples co-transfected with pGL4.26 ZFTA (− 3149 to +2) than in those co-transfected with pGL4.26 empty plasmid (Fig. 2A). As MCIDAS promotes the expression of other ciliary TFs^27^, we used a series of deletion constructs for pGL4.26 *ZFTA* (− 3149 to +2) to identify the MCIDAS-responsive element (Fig. 2B). We found that the MCIDAS-responsive luciferase activity in pGL4.26 *ZFTA* (− 220 to +2) was significantly reduced compared with that in pGL4.26 *ZFTA* (− 257 to  +2), suggesting the presence of MCIDAS-responsive element(s) in this region (Fig. 2B). In *Xenopus* embryonic epidermis, MCIDAS forms a tripartite complex with Dp1 and E2F4 or E2F5 to promote motile cilia formation, and E2F4 binds to sequences such as 5′-GGGCGGGAAA-3′ and 5′-AGAGCGCG-3′ to promote ciliogenesis^29^. As HeLa cells express Dp1 and E2Fs endogenously^30^, the overexpression of MCIDAS alone can result in the formation of this tripartite complex in HeLa cells. We found two sequences similar to these E2F4-binding sites, namely *ZFTA* (− 256 to − 249) and *ZFTA* (− 246 to − 239) (cyan letters in Fig. 2C). When these sequences were deleted or mutated (red letters in Fig. 2C), the MCIDAS-responsive luciferase activity was significantly reduced (Fig. 2D). These results suggest that *ZFTA* (− 256 to − 249) and *ZFTA* (− 246 to − 239) are MCIDAS-responsive elements that could contribute to promote *ZFTA* expression in the development of E1 cells and ZFTA-RELA expression in the pathogenesis of ST-EPN-RELA.

### ***Zfta***^***tm1/tm1***^ mice develop normally and are fertile

**Figure 3:**
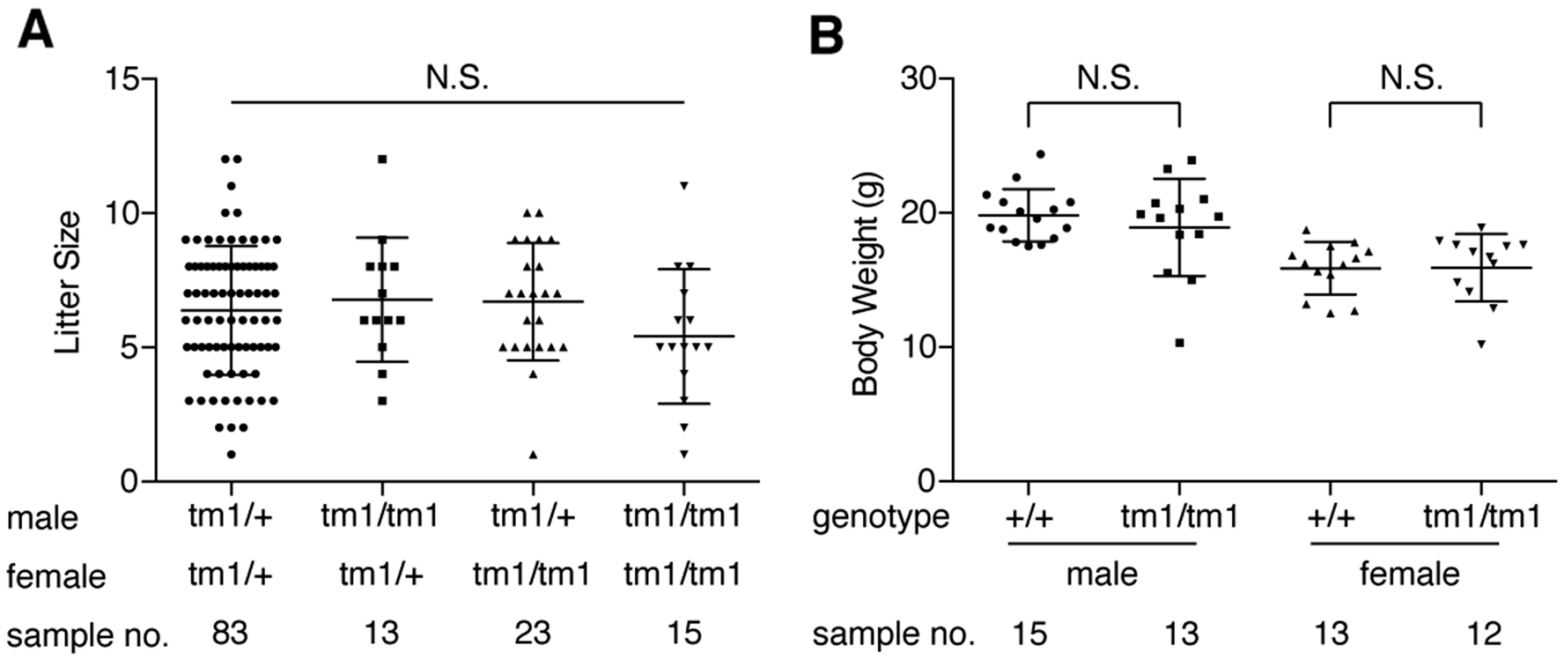
(**A**) Litter size of *Zfta*^*tm1/*+^ (tm1/+) and *Zfta*^*tm1/tm1*^ (tm1/tm1) breeding pairs. Data shown are the mean ± SD. Each point on the graph is the size of an individual litter. N.S., not significant (*p* = 0.35). (**B**) Body weight of WT (+/+) and *Zfta*^*tm1/tm1*^ (tm1/tm1) mice at P30–32. Data shown are the mean ± SD. Each point on the graph is the weight of an individual mouse. N.S., not significant (male, *p* = 0.41; female, *p* = 0.92).

Both human *ZFTA* and mouse *Zfta* consist of five exons (Ensembl GRCh38.p12 and GRCm38.p6, respectively). To address the potential role of *Zfta*, we used *2700081O15Rik*^*tm1(KOMP)Vlcg*^ (hereafter referred to as *Zfta*^*tm1*^) mice, in which a portion of the exon 3 and the whole exons 4 and 5 were replaced with *lacZ* gene. Zygotic *Zfta*^*tm1/tm1*^ mice, obtained from *Zfta*^*tm1/*+^ breeding pairs, were observed in the expected Mendelian ratio (Table 1), and they were viable for at least one year (n = 10 male and 13 female mice) and fertile (Table 1; Fig. 3A). At P31–34, body weights were similar between wildtype (WT) and *Zfta*^*tm1/tm1*^ mice (Fig. 3B). Paternal/zygotic and maternal/zygotic *Zfta*^*tm1/tm1*^ mice were also observed in the expected Mendelian ratio (Table1). Litter size was similar among the *Zfta*^*tm1/*+^ and zygotic *Zfta*^*tm1/tm1*^ breeding pairs (Fig. 3A). These results suggest that loss of *ZFTA* does not cause a major defect in the development, growth, or reproduction of mice.

**Table 1:** Numbers and percentages of P21-28 mice obtained from *Zfta*^*tm1*^ mutant mice breeding pairs.

paternal genotype	maternal genotype	*Zfta*^+*/*+^	*Zfta*^*tm1/*+^	*Zfta*^*tm1/tm1*^	Total	*p* value in *χ*^2^-test
*Zfta*^*tm1/*+^	*Zfta*^*tm1/*+^	130 (25.8%)	251 (49.9%)	122 (24.3%)	503	0.880
*Zfta*^*tm1/tm1*^	*Zfta*^*tm1/*+^	–	43 (48.9%)	45 (51.1%)	88	0.831
*Zfta*^*tm1/*+^	*Zfta*^*tm1/tm1*^	–	77 (50.0%)	77 (50.0%)	154	1.00
*Zfta*^*tm1/tm1*^	*Zfta*^*tm1/tm1*^	–	–	81 (100%)	81	–

### Normal morphology and protein expression in ***Zfta***^***tm1/tm1***^ E1 cells

**Figure 4:**
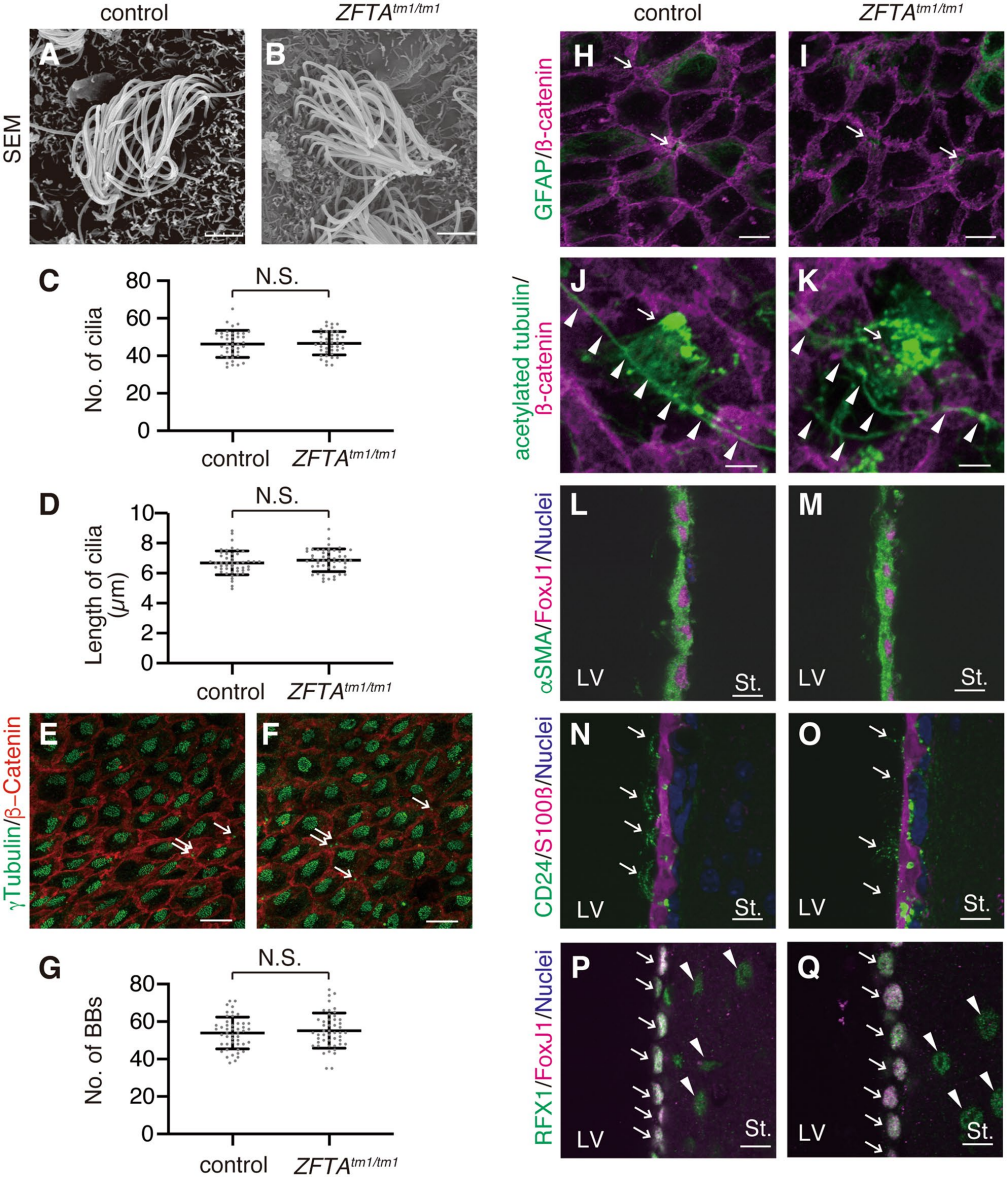
Normal development of E1 cells in *Zfta*^*tm1/tm1*^ mice. (A–D) Wholemount preparations of the lateral walls of the LV were observed by SEM in WT (**A**) and *Zfta*^*tm/tm*^ (**B**) mice. Bar = 2.5 µm. The number (**C**; n = 40 E1 cells from 4 control mice, n = 41 E1 cells from 4 *ZFTA*^*tm/tm*^ mice; *p* = 0.80) and length (**D**; n = 48 cilia from 4 control mice, n = 48 cilia from 4 *Zfta*^*tm/tm*^ mice; *p* = 0.28) of E1 cells’ motile cilia were quantified and plotted. Data shown are the mean ± SEM. Each point on the graph is the number of cilia in an individual E1 cell (**C**) and the length of an individual cilium (**D**). N.S., not significant. (**E**–**K**) Wholemount preparations of the lateral walls of LV at P30–35 were stained with antibodies against γ-tubulin (green in **E**, **F**), GFAP (green in **H**, **I**), acetylated tubulin (green in **J**, **K**) and β-catenin (red in **E**, **F**, magenta in **E**, **F**, **H**–**K**) in the control (**E**, **H**, **J**) and *Zfta*^*tm/tm*^ (**F**, **I**, **K**) mice. Bars = 10 µm (**H**, **I**, **L**–**Q**) and 40 µm (**I**, **K**). (**G**) The number of BBs was quantified in the WT (n = 48 E1 cells from 3 mice) and *Zfta*^*tm1/tm1*^ (n = 48 E1 cells from 3 mice, *p* = 0.50) E1 cells. Data shown are the mean ± SD. Each point on the graph is the number of BBs in an individual E cell. (**L**–**Q**) Coronal vibratome sections were stained with anti-αSMA (green in **L**, **M**), anti-FoxJ1 (magenta in **L**, **M**, **P**, **Q**), anti-CD24 antigen (green in **N**, **O**), anti-S100β (magenta in **N**, **O**) and anti-RFX1 (green in **P**, **Q**) antibodies in the control (**L**, **N**, **P**) and *ZFTA*^*tm1/tm1*^ (**M**, **O**, **Q**) mice at P30–35. Bar = 10 µm. The white arrows in **N** and **O** indicate the CD24-immunoreactive motile cilia of E1 cells. The white arrows and arrowheads in **P** and **Q** indicate the RFX1/FoxJ1-double positive E1 cells and RFX1-positive parenchymal cells, respectively. LV, lateral ventricle; St., striatum.

To address the effect of *Zfta* ablation on the development of E1 cells, we examined the morphology and protein expression in the control (WT and *Zfta*^*tm1/*+^) and *Zfta*^*tm1/tm1*^ E1 cells. The scanning electron microscopy observation of the control and *Zfta*^*tm1/tm1*^ E1 cells in the P31–35 wholemounts revealed that the morphology, number and length of motile cilia in E1 cells were similar in the control and *Zfta*^*tm1/tm1*^ mice (Fig. 4A–D). Wholemount staining for γ-tubulin, a protein that localises to basal bodies (BBs), and β-catenin, which localises to the intercellular junctions of B1 and E1 cells, showed that the number of BBs in the apical region of E1 cells at P31 was similar between the control and *Zfta*^*tm1/tm1*^ mice (Fig. 4E–G). In the LV walls, E1 cells surround the small apical surfaces of B1 cells, forming rosette-like structures called ‘pinwheels’^3^. In the control and *Zfta*^*tm1/tm1*^ wholemount preparation, B1 cells with single BB (arrows in Fig. 4E, F) were surrounded with E1 cells with approximately 50 BBs. Normal formation of the pinwheel structure in *Zfta*^*tm1/tm1*^ mice at P31 was confirmed through wholemount staining for GFAP, which localised in B1 cells’ apical endfoot (arrows in Fig. 4H, I), and β-catenin (Fig. 4H, I). A small group of neurons in the raphe extend supraependymal serotonergic axons to the V-SVZ^31^. In addition to motile cilia of E1 cells (arrows in Fig. 4J, K), anti-acetylated tubulin staining labelled the supraependymal axons in the control and *Zfta*^*tm1/tm1*^ V-SVZ wholemount preparations (arrowheads in Fig. 4J, K). These results suggest that the generation and apical docking of the BBs^32–34^ were unaffected in *Zfta*^*tm1/tm1*^ mice.

The injection of 4-hydroxy-tamoxifen into adult double transgenic mice with smooth muscle actin (*α*SMA)-driven CreER^T2^ and ROSA26-stop-enhanced yellow fluorescent protein transgenes (*αSMACreER*^*T2*^*::ROSA26*^*eYFP*^) resulted in the specific labelling of E1 cells in the forebrain^35^. We found that αSMA protein was expressed not only in the cytosol but also in motile cilia of FoxJ1-positive E1 cells in adult control mice, and that this staining pattern was similar in *Zfta*^*tm1/tm1*^ E1 cells at P31 (Fig. 4L, M). E1 cells express the calcium binding protein S100β^36^ and sialoglycoprotein CD24^37^. These proteins were similarly expressed in *Zfta*^*tm1/tm1*^ E1 cells at P31 compared with the control E1 cells (Fig. 4N, O). *RFX1* is expressed in human-induced pluripotent stem cell-derived optic progenitors (OPs), and it facilitates the differentiation of OPs into inner ear hair cell-like cells^38^. RFX1 is strongly expressed in the nuclei of the control FoxJ1-positive E1 cells at P31 and weakly expressed in some parenchymal cells in the striatum (arrows and arrowheads in Fig. 4P, respectively). This expression pattern is conserved in *Zfta*^*tm1/tm1*^ E1 cells (Fig. 4Q). These results indicate that E1 cells differentiate, develop motile cilia and become organised into pinwheels normally in *Zfta*^*tm1/tm1*^ mice.

### Normal planar cell polarity in ***Zfta***^***tm1/tm1***^ E1 cells

**Figure 5:**
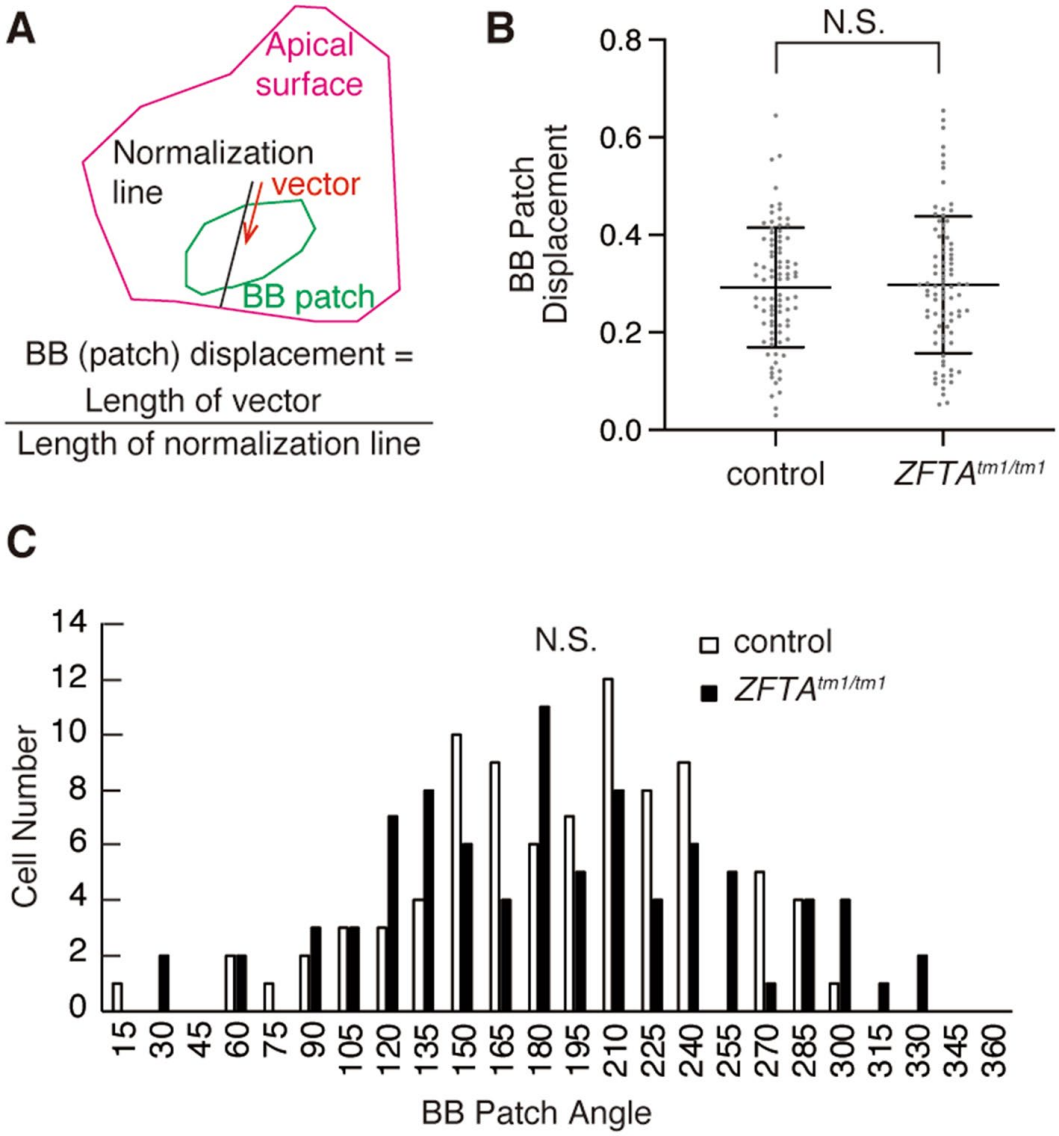
Normal translational polarity in *Zfta*^*tm/tm*^ E1 cells. (**A**) Schematic drawing showing the BB patch angle and BB patch displacement. A vector (red arrow) was drawn from the centre of the apical surface to that of the BB patch. To calculate the BB patch displacement, the length of the vector was normalised with the length of the line extended to the edge of the apical surface (black line). (**B**) Quantification of the BB displacement. Data shown are the mean ± SD. Control; n = 87 E1 cells, 3 mice: *Zfta*^*tm1/tm1*^; n = 86 E1 cells, 3 mice; *p* = 0.78. Each point on the graph is the BB displacement of an individual E1 cell. N.S., not significant. (**C**) Histogram showing the distribution of BB angles in the controls [white bars, circular standard deviation (CSD) = 57.19, n = 87 E1 cells, 3 mice] and *Zfta*^*tm1/tm1*^ (CSD = 69.534, n = 86 E1 cells from 3 mice) E1 cells at P30–35. N.S., not significant (*p* > 0.1).

The translational planar cell polarity of E1 cells is the asymmetric localisation of the cluster of BBs (BB patch) toward the downstream with regard to CSF flow in their apical area^39,40^. We drew vectors from the centre of the apical surface to the centre of the BB patch of E1 cells at P31 and measured the angles of these vectors (BB patch angle) and the displacement of the BB patch from the centre of the apical surface (BB displacement, Fig. 5A). Both the BB patch angle and BB patch displacement in the *Zfta*^*tm1/tm1*^ E1 cells were comparable with those in the control E1 cells (Fig. 5B, C). These results suggest that E1 cells are normally planar polarised in the brain ventricular epithelia.

### Normal ciliary beating of E1 cells, ependymal flow, ventricular volume and chain migration of new neurons in ***Zfta***^***tm1/tm1***^ mice

**Figure 6:**
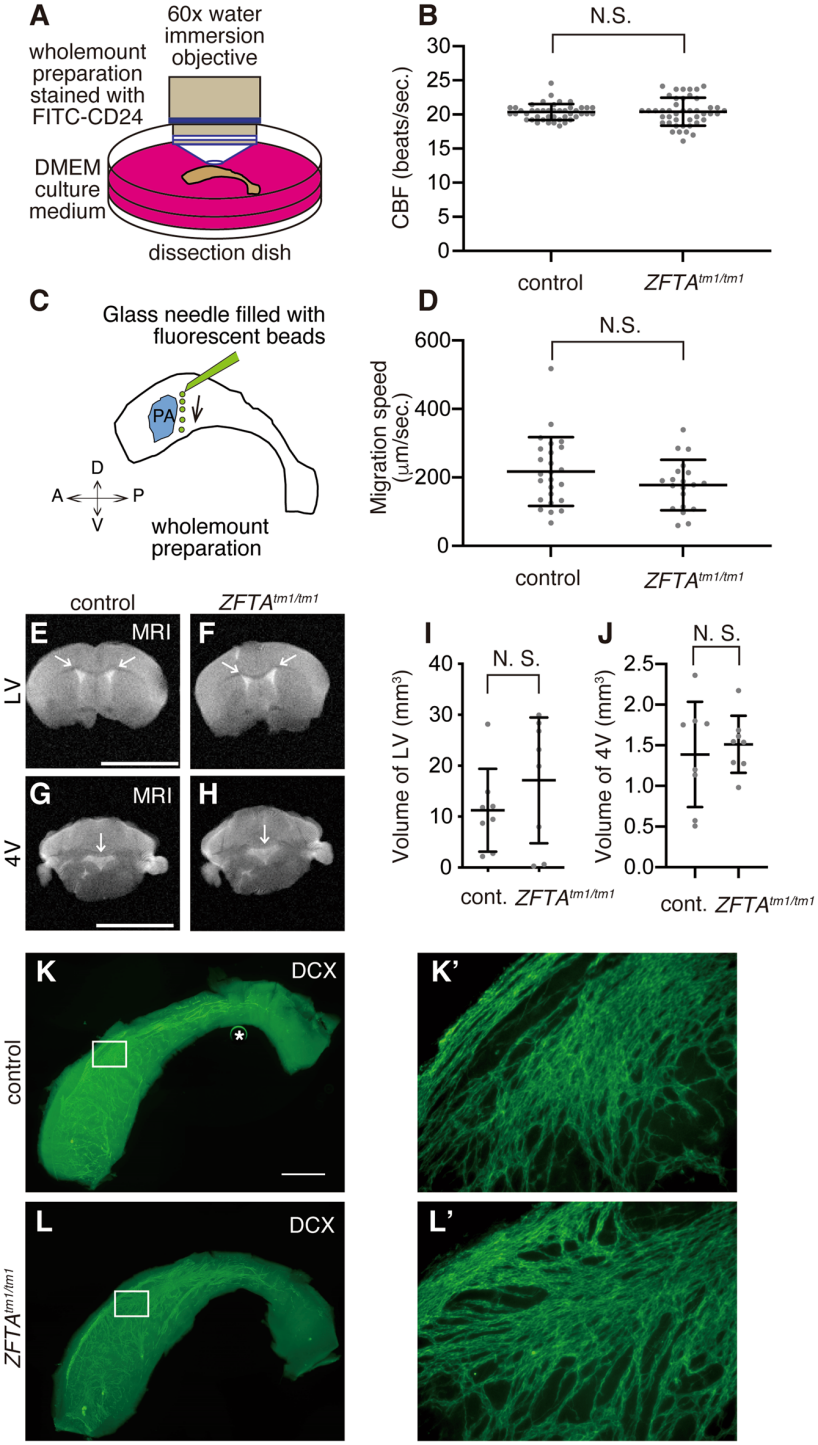
Normal ciliary beating and ependymal flow in *Zfta*^*tm1/tm1*^ mice. (**A**) Schematic of the live imaging of ciliary beating. Freshly isolated wholemount preparations of the lateral walls of the LV were stained with FITC-labelled CD24, washed with DMEM culture medium and stapled to a dissection dish filled with DMEM. Ciliary beating was observed with a water immersion objective and a high-speed camera. (**B**) The CBF was unaffected in *Zfta*^*tm1/tm1*^ mice at P31–35. Data shown are the mean ± SD. Forty-four cells from 4 control mice, 44 cells from 3 *ZFTA*^*tm1/tm1*^ mice, *p* = 0.89. N.S., not significant. Each point on the graph is the CBF of an individual E1 cell. See also Movies S1–S2. (**C**) Schematic of the ependymal flow assay. A glass needle was filled with fluorescent beads (green) and placed onto the wholemount preparations of the lateral walls of the LV. A, anterior; D, dorsal; P, posterior; PA, point of adhesion; V, ventral. (**D**) The speeds of the fluorescent beads on the wholemount preparation are expressed as the mean ± SD. Each point on the graph represents the speed of individual fluorescent beads. Control; n = 25 beads, 5 mice. *Zfta*^*tm1/tm1*^; n = 20 beads, 4 mice, *p* = 0.15. See also Movies S3–S4. (**E**–**H**) Coronal MRI scans showing the lateral (**E**, **F**) and fourth (**G**, **H**) ventricles in the control (**E**, **G**) and *Zfta*^*tm1/tm1*^ (**F**, **H**) mice at P31–35. White arrows indicate the lateral (**E**, **F**) and fourth (**G**, **H**) ventricles. Bar = 5 mm. (**I**, **J**) Quantification of the ventricular volumes in LV (**I**) and 4 V (**J**). Data shown are the mean ± SD. Each point on the graph represents the volume of ventricles in individual mice. Control (cont.); n = 8 mice: *Zfta*^*tm1/tm1*^; n = 8 mice; *p* = 0.28 (LV) and 0.64 (4 V). (**K**, **K**′, **L**, **L**′) Chain migration of new neurons is not affected in the *Zfta*^*tm1/tm1*^ V-SVZ. Migrating new neurons were stained with anti-DCX antibody in the control (**K**, **K**′) and *Zfta*^*tm1/tm1*^ (**L**, **L**′) wholemount preparations of the lateral walls of the LV at P31. The insets in K and L are magnified in **K**′ and **L**′, respectively. Bars = 1 mm. *Bubble in mounting media.

Sialoglycoprotein CD24 is expressed in the motile cilia of E1 cells^41,42^. To visualise the beating of motile cilia in E1 cells, we marked it with fluorescein isothiocyanate (FITC)-labelled CD24 antibody and observed it in *en face* view (Fig. 6A). The quantification of the ciliary beating frequency (CBF, strokes/sec) in E1 cells from the *Zfta*^*tm1/tm1*^ and control mice at P31–35 revealed that the ablation of *Zfta* did not affect the CBF in E1 cells (Fig. 6B, Movies S1, S2). The continuous beating of cilia on the apical surface of E1 cells generates a unidirectional fluid flow called ependymal flow^1,2,43^. To visualise the ependymal flow, we placed polystyrene latex fluorescent microbeads on live preparations of the lateral wall of the LV from control and *Zfta*^*tm1/tm1*^ mice (Fig. 6C)^44^. When a small number of microbeads were placed in the posterior dorsal region of the LVs in control mice, a dorsal to ventral current was observed (Movie S3). In the wholemount preparations of *Zfta*^*tm1/tm1*^ mice, the overall directionality of this flow was similar to that in the controls (Movie S4). The speed of the fluorescent beads was not significantly different between the control and *Zfta*^*tm1/tm1*^ wholemounts (Fig. 6D). Defective ciliary beating results in the accumulation of CSF and consequently hydrocephalus^45^. The volumes of LV and fourth ventricle (4 V) in *Zfta*^*tm1/tm1*^ mice at P31 were similar to those in the control mice (Fig. 6E–J). New neurons generated in the V-SVZ migrate as chains anteriorly toward the olfactory bulb^46^. E1 cells help guide the chain migration by generating a gradient of chemorepulsive Slit2 through the beating of their motile cilia^5^. Wholemount staining for doublecortin (DCX)-positive new neurons revealed that the chain migration of new neurons in the LV walls was unaffected in *Zfta*^*tm1/tm1*^ mice at P31 (Fig. 6K, K′, L, L′). Altogether, these results suggest that the ciliary beating of E1 cells and ependymal flow are largely normal in *Zfta*^*tm1/tm1*^ mice.

### ZFTA-RELA-responsive elements upstream of human *ZFTA*

**Figure 7:**
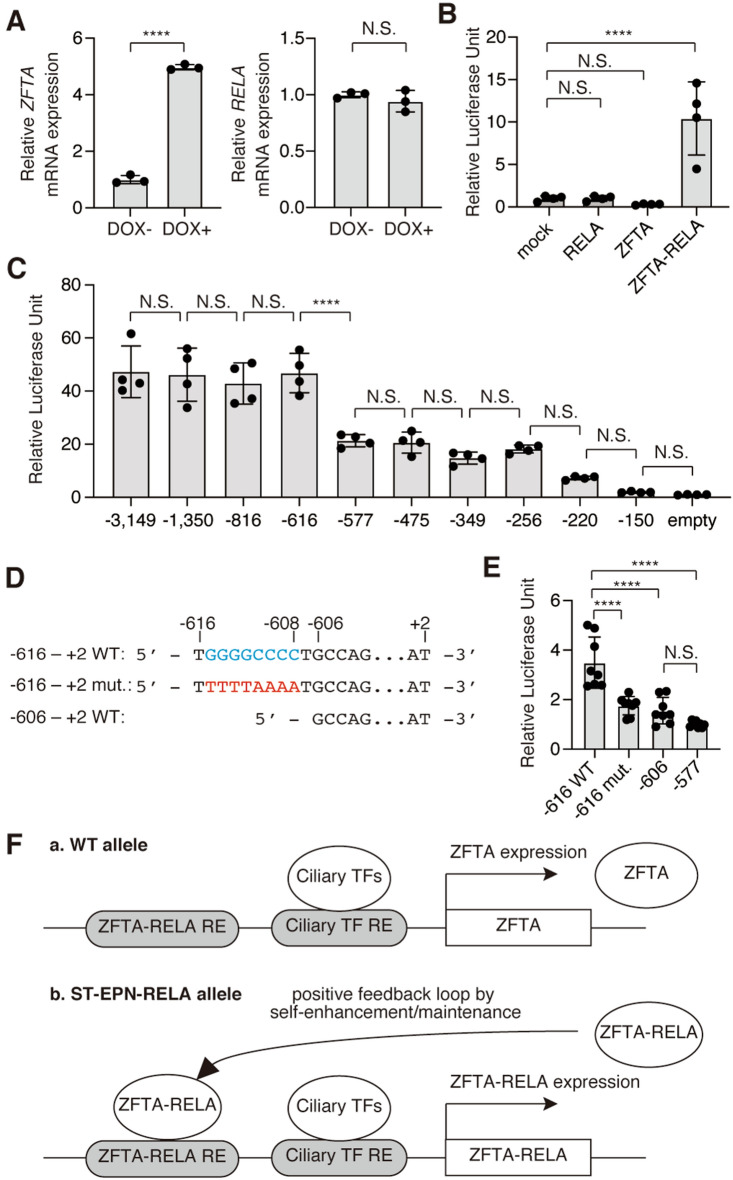
ZFTA-RELA-responsive element upstream of *ZFTA*. (**A**) *ZFTA* (left) and *RELA* (right) mRNA expression levels in doxycycline-inducible ZFTA-RELA expressing HEK293 cells. Cells were cultured in the absence (DOX−) or presence (DOX+) of doxycycline overnight, and the mRNA levels of *ZFTA* and *RELA* were quantified by qPCR. Data shown are the mean ± SD. Each point on the graph is the relative mRNA level of an individual sample. N = 3 each. ****p < 0.0001; N.S., not significant. Note that the primer set used to amplify *RELA* cDNA targets the 3′ UTR of *RELA*, so it amplifies endogenous *RELA*, but not exogenous *ZFTA-RELA*. (**B**) Luciferase assay upon transfection of HeLa cells with mock, *RELA*, *ZFTA* and *ZFTA-RELA* expression plasmids. Luciferase activities were normalized by the mean of the activity obtained in cells co-transfected with pGL4.26 *ZFTA* (− 3149 to + 2) and mock plasmids. Data shown are the mean ± SD. N = 4 each. ****p < 0.0001; N.S., not significant. (**C**) Luciferase assay upon transfection of HeLa cells with ZFTA-RELA expression plasmids and the series of deletion mutants for ZFTA’s upstream sequence used in Fig. 2B. Luciferase activities were normalized by the mean of activity obtained in cells transfected with pGL4.26 empty plasmid. The labels on the graphs indicate only the start of the ZFTA upstream sequence. For example, pGL4.26 ZFTA (− 3149 to + 2) is labeled as “− 3149". Data shown are the mean ± SD. N = 4 each. ****p < 0.0001; N.S., not significant. (**D**) Schematic diagram of the constructs used in Fig. 7E. Cyan and red letters indicate WT and mutant sequences, respectively. (**E**) Luciferase assay upon transfection of HeLa cells with ZFTA-RELA expression plasmids and the constructs shown in Fig. 7D. Luciferase activities were normalized by the mean of activity obtained in cells transfected with pGL4.26 *ZFTA* (− 577 to +2), which is indicated as “− 577”. Each point on the graph is the relative luciferase activity of an individual sample. N = 8 each. *, ****p < 0.0001; N.S., not significant. (**F**) Models of the ZFTA expression in the WT allele (**a**) and ZFTA-RELA expression in the ST-EPN-RELA allele (**b**). RE, responsive element.

Consistent with a previous report in 293 T cells^18^, the overexpression of ZFTA-RELA promoted the expression of endogenous *ZFTA* mRNA in doxycycline-inducible ZFTA-RELA expressing cells established from HEK293 cells (Fig. 7A left). However, ZFTA-RELA did not affect the expression of *RELA* mRNA (Fig. 7A right). In the luciferase assay using pGL4.26 *ZFTA* (− 3149 to +2) and HeLa cells, ZFTA-RELA increased the luciferase activity, while ZFTA or RELA did not (Fig. 7B). These results raise the possibility that ZFTA-RELA promotes its expression through its own responsive element(s) present in the 5′ upstream sequence of *ZFTA*. Using the series of deletion constructs for pGL4.26 *ZFTA* (− 3149 to +2), we narrowed down the candidate ZFTA-RELA-responsive element (Fig. 7C). There were statistically significant differences between pGL4.26 *ZFTA* (− 616 to +2) and pGL4.26 *ZFTA* (− 577 to +2), suggesting the presence of ZFTA-RELA responsive sequences in this region. In 293 T cells, ZFTA-RELA promotes the expression of downstream genes by binding to sequences called MEME-1, -2 and -3, whereas in cultured cells derived from mouse ST-EPN-RELA, ZFTA-RELA binds only to MEME-2 (5′-GT/GGGCCCC-3′)^18^. The region from − 616 to − 577 contained a MEME-2-like sequence (cyan in Fig. 7D). Deletion or mutation in this region (red in Fig. 7D) significantly reduced the ZFTA-RELA-responsive luciferase activity (Fig. 7E), suggesting that this region functions as a ZFTA-RELA-responsive sequence.

## Discussion

In the present study, we found that the ependymoma-associated *Zfta* gene was expressed in pre-E1 cells, which are the source of ependymomas^10^, possibly under the control of ciliary TFs (Fig. 7Fa). Since the expression of *ZFTA-RELA* fusion gene is likely regulated by the 5′ upstream sequence of *ZFTA*^18^, these TFs could be involved in the expression of oncogenic ZFTA-RELA in pre-E1 cells, potentially underlying the pathogenesis of ST-EPN-RELA. As the ZFTA-RELA-responsive sequence was found in the *ZFTA* upstream sequence^12,18^ (Fig. 7), we propose the following model as a possible mechanism for ZFTA-RELA expression: ciliary TFs, including MCIDAS, initiate ZFTA-RELA expression, which in turn promotes its own expression, forming a positive feedback loop (Fig. 7Fb). However, the present study provides only indirect evidence by luciferase assay, and it will be necessary to investigate the direct involvement of ciliary TFs and ZFTA-RELA by other methods such as chromatin immunoprecipitation in the future. We also characterised *Zfta*^*tm1/tm1*^ mice by replacing a part of *Zfta* with *lacZ*. Although we did not observe an apparent abnormality in *Zfta*^*tm1/tm1*^ mice in physiological conditions in a C57BL/6N-background, *Zfta* could play an important role in pathological conditions and/or in another genetic background. As the loss of *Zfta* may have been compensated for by other genes, the identification of such genes is necessary to elucidate the function of ZFTA in vivo. Given that loss of *Zfta* does not significantly affect ependymal cell development or function, inhibiting the function of the ZFTA portion of ZFTA-RELA in ST-EPN-RELA may be a therapeutic strategy with fewer adverse effects.

In the present study, we showed that ZFTA localizes to the nuclei in HeLa and pre-E1 cells. As ZFTA is predicted to have four zinc finger domains^18^, which interact with DNA^47^, it could function as a transcriptional regulator. ZFTA would require some co-factor for transcriptional regulation, as a single overexpression of ZFTA in mouse neural stem cells does not alter the gene expression status^12^. The fusion of ZFTA with MAML2, MKL2, RelA and YAP1 is found in chondroid lipoma and ependymoma^11–15,20^. MKL2, RelA and YAP1 function as transcriptional regulators, and their nuclear translocation is a key step for their transcriptional activity^48–50^. The constitutive nuclear localisation of ZFTA-RELA has been demonstrated to cause the hyperactivation of NF-κB signalling in ST-EPN-RELA^12^. The molecular mechanisms underlying the nuclear localisation of ZFTA are of prime importance to understand the pathogenesis of chondroid lipoma and ST-EPN-RELA.

We demonstrated in wholemount preparations of the mouse LV that the expression of *Zfta* mRNA reached its peak on E17.5 and that pre-E1 cells expressed Zfta protein. Consistent with our observation, single-cell transcriptional profiling revealed that *Zfta* mRNA was expressed in *αSMACreER*^*T2*^*::tdTomato* and Sox2-GFP-double positive E1 cells isolated from 8-week-old mice^35^. However, in our preliminary immunohistochemistry study using 4- and 8-week-old mouse brains, Zfta immunoreactivity was under the detection level (data not shown). This discrepancy could be due to the affinity of our antibody raised against human ZFTA (563^rd^–592^nd^ AA), which is 67% (20/30) identical to mouse Zfta (Fig. S1). Antibodies recognising mouse Zfta with higher affinity would be required to further analyse the expression profile of Zfta.

In the *Zfta*^*tm1*^ mice used in the present study, a portion of exon 3 and whole exons 4 and 5 were replaced with *lacZ* (UC Davis KOMP Repository Knockout Mouse Project: https://www.komp.org/index.php). This knock-in mouse strain could express a fusion protein of Zfta (from the 1st to 273rd AA out of 678 AA, including its first zinc finger domain and coiled-coil domain) and full-length β-galactosidase. Thus, we cannot exclude the possibility that the expression of ZFTA-β-galactosidase fusion protein compensated for the loss of full-length Zfta in *Zfta*^*tm1/tm1*^ mice. We speculate that this is unlikely because in our preliminary study, we could not detect signals in X-gal staining in *Zfta*^*tm1/tm1*^ mice (data not shown). The artificial *Zfta-lacZ* fusion gene could be silenced by the methylation of CpG islands in the regulatory elements of *Zfta*, as the genomic region of *ZFTA* is highly GC-rich (77% in 1 kbp upstream). Nevertheless, analysis of the phenotype of mouse lines lacking the entire *Zfta* remains an important issue for the future.

We found that smooth muscle actin *α*SMA was expressed in the cytosol and motile cilia of E1 cells. Various signalling molecules are concentrated in primary cilia, which function as a signalling centre^51,52^. Actin network in the primary cilia is required for the release of ectosomes, which contain activated G protein-coupled receptors from the ciliary tip and contribute to the downregulation of ciliary signalling^53^. As chemoreceptors, such as bitter taste receptors and the sonic hedgehog receptor Patched, are found in motile cilia in the trachea^54,55^, *α*SMA in the motile cilia of E1 cells may also contribute to the ectocytosis of ciliary receptors and the downregulation of ciliary signalling.

RFX family TFs regulate ciliogenesis and differentiation. The ablation of *Rfx3* in mice causes defective ciliogenesis in node and ependymal cells, resulting in left–right asymmetry defects and hydrocephalus, respectively^56,57^. The differentiation of β-cells was also affected in *Rfx3* knockout mice^58^. The conditional knockout of Rfx1 and Rfx3 in inner ear hair cells using *Gfi1*-Cre mice causes the degeneration of cilia^59^. In the present study, we found that Rfx1 was expressed in E1 cells and unidentified parenchymal cells in the striatum (Fig. 5) and other brain regions (data not shown). The identity of Rfx1-positive parenchymal cells in the striatum and the role of Rfx1 in the ciliogenesis and differentiation of E1 and parenchymal cells are interesting issues to examine. The expression of RFX2 and FOXJ1 in ependymomas is higher than that in normal tissues and other brain tumors such as astrocytomas^60,61^. As ciliary TFs including RFX family promoted luciferase activity using the upstream sequence of *ZFTA*, these TFs might be involved in the expression of ZFTA-RELA in ST-EPN-RELA. Analysis of the expression of ciliary TFs in ST-EPN-RELA and elucidation of the regulatory mechanism of ZFTA-RELA expression are important issues to be addressed.

Our study revealed a previously unknown profile and mechanism of *ZFTA* expression and the effects of its loss-of-function in vivo. This study can help elucidate not only the development of E1 cells but also the pathogenesis of ST-EPN-RELA.

## Materials and methods

### Ethics declaration

All animal experiments were approved by the institutional animal care and use committees of the University of Tokyo and Musashino University and conducted in accordance with institutional guidelines. The study is reported in accordance with ARRIVE guidelines (https://arriveguidelines.org).

### Animals

All mice were housed in a specific pathogen-free environment at 22–25 °C and 50–70% humidity in a 12 h light/12 h dark cycle in autoclaved cages containing Paper Clean (SLC) and Enviro-Dri Environmental Enrichment (SSP) with free access to sterile foods (Oriental Yeast, MF) and filtered water (AION, Kanefiel FD-005, 5 µm). Shepherd Shack Environmental Enrichment (SSP) was added to the cages of breeding pairs. The *Zfta*^*tm1*^ mouse strain (UC Davis KOMP Repository, VG12886) was generated using embryonic stem cell clone 2700081O15Rik_AA12, in which a portion of exon 3 and whole exons 4 and 5 were replaced with *lacZ* and a floxed neomycin cassette^62^ and reanimated from cryopreserved sperm in RIKEN BioResource Centre. After mating the reanimated mouse with C57BL/6NJcl WT mice, its offspring were mated with FVB/N-Tg(EIIa-cre)C5379Lmgd/J mice (Jackson Laboratory, 003314) to remove the floxed neomycin-resistance gene cassette. *ZFTA*^*tm1*^::FVB/N-Tg(EIIa-cre)C5379Lmgd/J mice were mated with C57BL/6NJcl WT mice to remove the *EIIa-cre* transgene. After these mating, *Zfta*^*tm1*^ were kept in bred. Genotyping was performed by genomic PCR using SapphireAmp Fast PCR Master Mix (TaKaRa) and the following primers: WT allele, TDF2 (5′-GTG GAT GGG TCG GAG CAA CTG-3′) and TDR2 (5′-CGC GTC GCC TGG AGA AGA AC-3′); *tm1* allele, SU (5′-GCT AGG CAG AGA TCA GCC AC-3′) and LacZ Rev (5′-GTC TGT CCT AGC TTC CTC ACT G-3′); and Cre allele, Cre F (5′-GGA CAT GTT CAG GGA TCG CCA GGC G-3′) and Cre R (5′-GCA TAA CCA GTG AAA CAG CAT TGC TG-3′). Body weight was scaled using a GX-2000 (A&D). Both males and females were used for all experiments.

### qPCR

The LV walls at the indicated developmental time points were micro-dissected as previously described^41,42^. Total RNAs and cDNAs were prepared as previously described^63^. qPCR was performed using Thunderbird SYBR qPCR Mix (TOYOBO) on a StepOne system (Roche) or Light Cycler 96 (Nippon Genetics). The relative mRNA expression level was determined by the 2^−∆∆C*t*^ method using the *TATA box binding protein* (*Tbp*) as an endogenous control to normalise the samples^64,65^. The following primers were used: mouse *Zfta* F, 5′-GGG TCT GGA GGA AGA GAT GCC-3′; mouse *ZFTA* R, 5′-TGC CCT CTG CTT TCC CAC TCC-3′; mouse *Tbp* F, 5′-ACC CTT CAC CAA TGA CTC CTA TG-3′; mouse *Tbp* R, 5′-TGA CTG CAG CAA ATC GCT TGG-3′; human *ZFTA* F, 5′-GGG TCT GGA GGA AGA GAT GCC-3′; human *ZFTA* R, 5′-TGC CCT CTG CTT TCC CAC TCC-3′; human *RELA* 3′ untranslated region (UTR) F, 5′-CCT AGA GAC AGA AGC AGG CTG G-3′; human *RELA* 3′ UTR R, 5′-CTC AAA CGC TGG TGT TAG GCA C-3′; human *TBP* F (PrimerBank ID 285026518c3) 5′-GAGCCAAGAGTGAAGAACAGTC-3′; human *TBP* R, 5′-GCTCCCCACCATATTCTGAATCT-3′.

### Cell culture, transfection, immunocytochemistry, and immunoblotting

Cells were cultured in Dulbecco’s Modified Eagle Medium (Nacalai Tesque) supplemented with 10% foetal bovine serum (Biowest) and an antibiotic–antimycotic solution (Nacalai Tesque) at 37 °C in a 5% CO_2_ condition overnight. A cell line expressing Flag-tagged ZFTA-RELA in a doxycycline-inducible manner was established using the NF-κB reporter-HEK293 cell line (BPS Bioscience) and Tet-One Inducible Expression System (Clontech). Transfection was performed using Fugene 6 [Promega, for immunocytochemistry (ICC)] and HilyMax (Dojindo, for immunoblotting and luciferase assay) as described previously^66^.

For ICC, transfected HeLa cells were washed with Dulbecco’s phosphate-buffered saline (PBS), fixed with 4% paraformaldehyde (PFA) dissolved in 100 mM phosphate buffer (PB, pH7.4) at room temperature (RT) for 10 min, washed with PBS, incubated in 10% normal goat serum (EMD Millipore) and 0.2% Triton X-100 (Sigma-Aldrich) in PBS (hereafter referred to as the blocking buffer), incubated with primary antibodies (listed in the ‘Antibodies’ section) diluted in the blocking buffer at 4 °C overnight or at RT for 1 h, washed with PBS, incubated with secondary antibodies conjugated with fluorescent dyes (Abcam) and DRAQ5 (Biostatus) in the blocking buffer at RT for 1 h, washed with PBS and mounted with Aqua-Poly/Mount (Polysciences). Confocal microscopy images were acquired using an FV1000 (Olympus) or 710META (Zeiss).

For immunoblotting, the transfected HeLa cells were washed with PBS, lysed and sonicated in Laemmli sample buffer and subjected to sodium dodecyl sulfate–polyacrylamide gel electrophoresis. The separated proteins were transferred to Immobilon-P membranes (0.45 µm, Millipore). After blocking nonspecific binding sites with Blocking One (Nacalai Tesque), the membranes were incubated with primary antibodies (listed in the ‘Antibodies’ section), washed with Tris-buffered saline (TBS, pH7.5) with 0.2% (v/v) Tween-20 (wash buffer), incubated with secondary antibodies conjugated with horseradish peroxidase (HRP, Jackson ImmunoResearch) and washed with the wash buffer. Immunosignals were visualised using Chemi-Lumi One (Nacalai Tesque) or Immobilon Western Chemiluminescent HRP Substrate (Millipore) and captured with a Fusion SL (Vilber-Lourmat).

### Immunohistochemistry and wholemount staining

After deeply anesthetised with inhalation of sevoflurane (Fujifilm), adult mice were transcardially perfused with PBS, followed by 4% PFA in 100 mM PB (pH7.4). The embryos were euthanised through rapid cervical dislocation using sharp scissors without perfusion. Adult and embryonic brains were dissected and fixed with 4% PFA at 4 °C overnight and rinsed with PBS. Vibratome sections (50 µm thick) and cryosections (12 µm thick) were prepared using Neo-LinearSlicer NLS-MT (Dosaka EM) and CN3050S (Leica), respectively. The sections were stained as described previously^41,42^ using antibodies listed in the ‘Antibodies’ section. Wholemounts of the lateral walls of the LV were prepared and stained as described previously^44^. Fluorescent images were observed using a BZ-X710 all-in-one fluorescence microscope (Keyence), LSM710 (Zeiss) or FV1000 (Olympus). The BB patch angle and displacement were analysed using ImageJ software (National Institute of Health) as described previously^41,42^. The results of each experiment were based on studies of three or more mice per group. If necessary, the brightness and contrast of the images were adjusted using ImageJ software in accordance with the journal’s guideline.

### Antibodies

The primary antibodies used were as follows: mouse anti-β-catenin (BD Biosciences, 610153), rabbit anti-β-catenin (Sigma-Aldrich, C2206), rabbit anti-C11orf95/ZFTA (Abcam, ab170283), rat anti-CD24 conjugated with FITC (Thermo Fisher, 11-0242-82), rabbit anti-Doublecorin (DCX, cell signalling, #4604), mouse anti-FoxJ1 (eBiosciences, 14-9965), mouse anti-Glyceraldehyde-3-phosphate dehydrogenase (GAPDH, Millipore, MAB374), chicken anti-GFAP (Aves, GFAP), rabbit anti-RFX1 (Novus, NBP1-52654), rabbit anti-S100β (Proteintech, 15146-1-AP), rabbit anti-smooth muscle actin (*α*SMA, Proteintech, 55135-1-AP), mouse anti-acetylated tubulin (Sigma-Aldrich, T6793) and rabbit anti-γ-tubulin (Sigma-Aldrich, T5192). The secondary antibodies used were as follows: conjugated to Alexa Fluor dyes (Abcam) or HRP (Jackson ImmunoResearch).

### Scanning electron microscopy

Wholemount preparations of the lateral wall of the LVs were fixed with 2% PFA and 2.5% glutaraldehyde (Nacalai Tesque) in PB at RT for 1 h, washed with PB, postfixed with 1% osmium tetroxide (Electron Microscopy Sciences) in PB for 45 min at RT, rinsed with deionised water and dehydrated first in ethanol then with CO_2_ using the critical point drying method. The samples were coated with gold/palladium alloy by sputter coating. The surface of the lateral wall was examined under a Hitachi S-4800 scanning electron microscope using Quantax 400 software (Bruker) for image acquisition.

### Luciferase assay

The upstream sequence of *ZFTA* from − 3149 to +2 (corresponding to the second nucleotide of *ZFTA*’s first codon) was amplified by PCR using a human bacterial artificial chromosome clone (RP11-179P19, Advanced GenoTechs), which lacks the − 154th guanine base, as a template, and cloned into a pGL4.26 (luc2/minP/Hygro) vector (Promega). A series of deletion mutants for this plasmid was generated by PCR using PrimeSTAR MAX or Tks Gflex DNA polymerase (TaKaRa). HeLa cells were transfected with the indicated plasmids listed in Table S1 using HilyMax (Dojindo) and incubated overnight. Luciferase assay was performed using TriStar LB 941 (Berthold) and the following reaction buffers: 100 mM Tris–HCl (pH7.8, Nacalai Tesque), 5 mM MgCl_2_ (Wako Pure Chemical), 250 µM Coenzyme A trilithium salt (Oriental Yeast), 150 µM adenosine triphosphate (Nacalai Tesque), 150 µg/mL D-Luciferin potassium salt (Cayman Chemical), 0.5 mM dithiothreitol (Nacalai Tesque), 50 µM ethylenediaminetetraacetic acid (pH8.0, Dojindo) and 0.1% Triton X-100 (Sigma-Aldrich).

### High-speed live imaging of ciliary beating and ependymal flow assay

High-speed live imaging of ciliary beating and ependymal flow assay were performed as described previously with minor modifications^41,42^. For the high-speed imaging of ciliary beating, the wholemount preparations of the lateral walls of the LVs were incubated with FITC-labelled rat anti-CD24 antibody (BD Biosciences) in DMEM (Nacalai) for 30 min at RT, rinsed with DMEM, placed on a dissection dish, and fixed with staples. Ciliary beating was recorded with a 10 ms exposure time at 100 frames per second (fps) at RT using an Olympus BX53 microscope, LUMFLN60XW water immersion objective lens (NA 1.10), ORCA-Flash4.0 V3 high-speed camera (HAMAMATSU) and high-speed recording (HSR) software (HAMAMATSU).

For the ependymal flow assay, a glass micropipette filled with fluorescent polystyrene latex microbeads (2 µm, Polysciences) attached to an MO-10 micromanipulator (Narishige) was lowered onto the wholemount, and the microbeads were deposited onto the ventricular surface. The movement of microbeads was recorded at RT at 20 fps using an Olympus SZX16 fluorescent dissection microscope, ORCA-Flash4.0 V3 high-speed camera and HSR software. The speeds of the migrating fluorescent beads were quantified using the Manual Tracking plugin for ImageJ software (written by Dr. Fabrice P. Cordelieres).

### Magnetic resonance imaging (MRI)

Axial image sequences of fixed brains were acquired using a 7 T MR scanner (Unity Inova system; Agilent Technologies) and a handmade proton surface coil (repetition time/echo time = 4200/36 ms, 10 × 10 mm, thickness = 0.5 mm, slice gap = 0 mm). The sizes of the ventricles were analysed using ImageJ software.

### Statistical analysis

All results shown in the dot plot and bar graphs are expressed as mean ± SEM. Means of two groups, three or more groups with one variable, and three or more groups with two variables were compared with two-tailed Student's *t* test, one-way analysis of variance (ANOVA) with Tukey’s post-hoc test, and two-way ANOVA with Šidák’s post-hoc test, respectively, using Prism 9 (GraphPad). The proportion of *Zfta*^*tm1*^ mice was compared to expected Mendelian ratios by the chi-square test using Excel (Microsoft). Distributions of angles were compared with Watson’s *U*^*2*^ test using Oriana software (Kovach Computing Services). Differences were considered significant at *p* < 0.05.

## Supplementary Information

Supplementary Video S1.

Supplementary Video S2.

Supplementary Video S3.

Supplementary Video S4.

Supplementary Tables.

Supplementary Figure S1.

Supplementary Figure S2.

Supplementary Legends.
