# Supplementary material for: Ependymoma associated protein Zfta is expressed in immature ependymal cells but is not essential for ependymal development in mice
Source: Sci Rep. 2022 Jan 27;12:1493. doi: 10.1038/s41598-022-05526-y (PMC8795269; doi:10.1038/s41598-022-05526-y)
Supplement: Supplementary file 5 — Supplementary Tables. [file 41598_2022_5526_MOESM5_ESM.docx]

Table S1. Plasmids used in the present study

| gene | NCBI Reference Sequence | backbone vector | obtained from |
| --- | --- | --- | --- |
| C11orf95 (human ZFTA) | NM_001144936 | pCMV6-ENTRY | ORIGENE |
| 2700081O15Rik  (mouse Zfta) | NM_175381.6 | pcDNA3.1 | GenScript |
| E2F4 | NM_001950.3 | pKA1U5 | RIKEN BRC |
| E2F5 | NM_001951.3 | pKA1U5 | RIKEN BRC |
| FOXJ1 | NM_001454 | pCMV-SPORT6 | RIKEN BRC |
| GMNC | NM_001146686.2 | pcDNA3.1-C-(k)DYK | GenScript |
| MCIDAS | NM_001190787.1 | pcDNA3.1-C-(k)DYK | GenScript |
| MYB | NM_002918 | pCMV-SPORT6 | RIKEN BRC |
| RFX1 | NM_002918 | pCMV-SPORT6 | RIKEN BRC |
| RFX2 | NM_000635.3 | pcDNA3.1-C-(k)DYK | GenScript |
| RFX3 | NM_134428.2 | pcDNA3.1-C-(k)DYK | GenScript |
