# Supplementary figures and images for: Ependymoma associated protein Zfta is expressed in immature ependymal cells but is not essential for ependymal development in mice
Source: Sci Rep. 2022 Jan 27;12:1493. doi: 10.1038/s41598-022-05526-y (PMC8795269; doi:10.1038/s41598-022-05526-y)

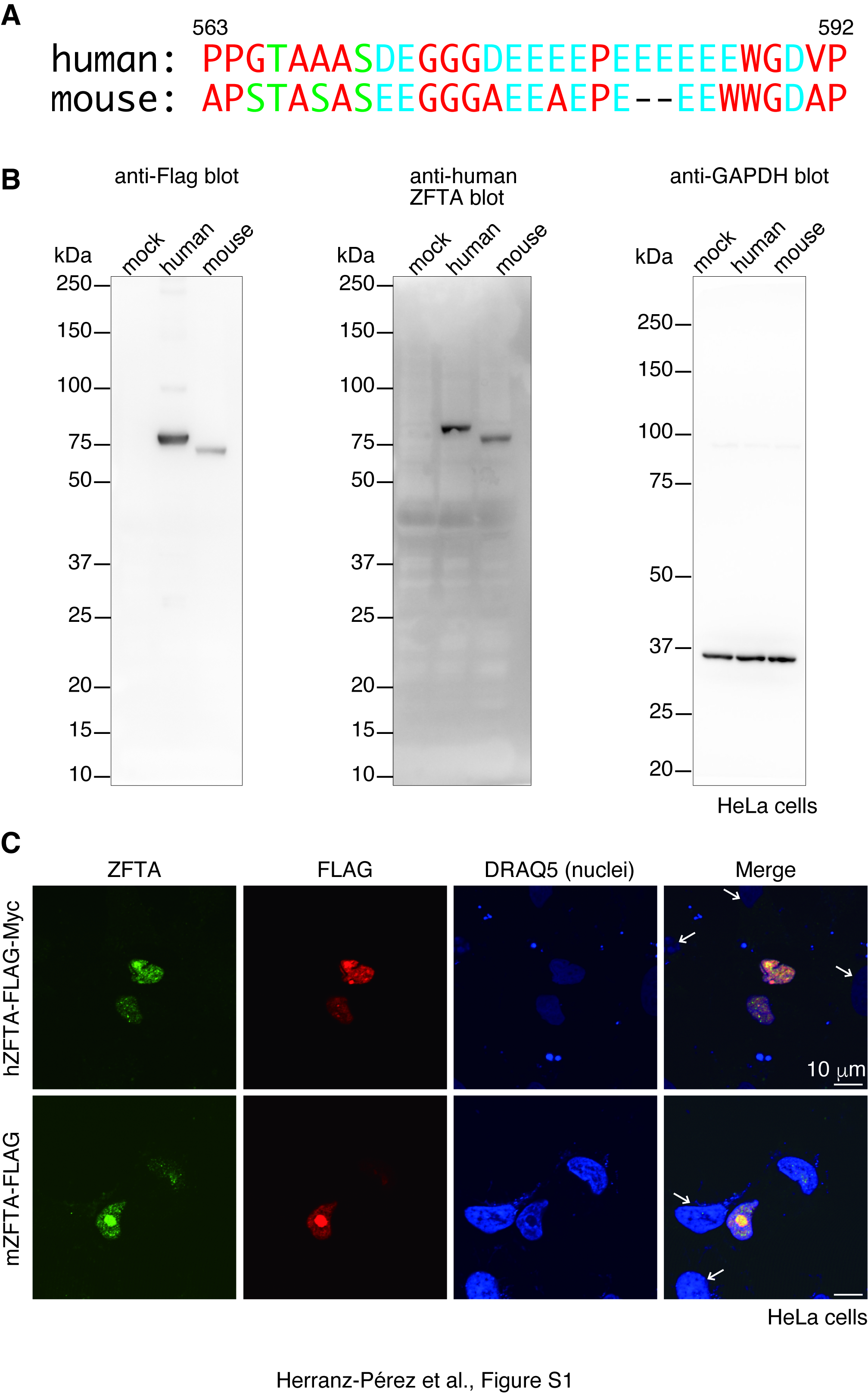

Supplement: Supplementary file 6 — Supplementary Figure S1. [file 41598_2022_5526_MOESM6_ESM.tif]

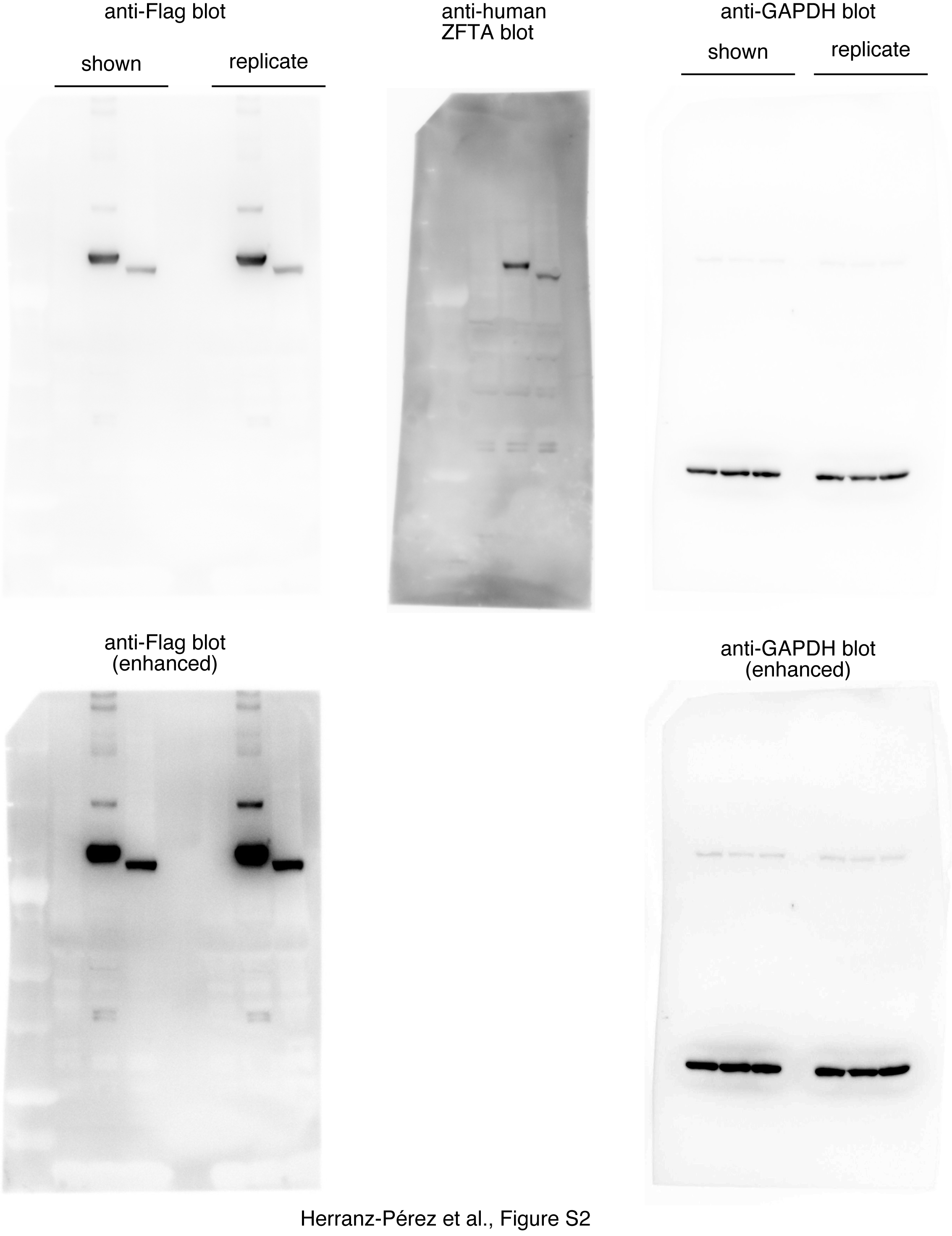

Supplement: Supplementary file 7 — Supplementary Figure S2. [file 41598_2022_5526_MOESM7_ESM.tif]
