# Supplementary material for: Ependymoma associated protein Zfta is expressed in immature ependymal cells but is not essential for ependymal development in mice
Source: Sci Rep. 2022 Jan 27;12:1493. doi: 10.1038/s41598-022-05526-y (PMC8795269; doi:10.1038/s41598-022-05526-y)
Supplement: Supplementary file 8 — Supplementary Legends. [file 41598_2022_5526_MOESM8_ESM.docx]

**Figure S1. Specificity of an anti-human C11orf95/ZFTA antibody**

(A) The epitope of anti-human C11orf95/ZFTA antibody used in this study (from the 563^rd^ to 592^nd^ amino acids (AAs) of human ZFTA) is 67% (20/30) identical to mouse Zfta. Aliphatic or cyclic nonpolar (A,G,P,V,W), acidic polar (D,E) and hydroxyl-containing polar (S,T) AAs are shown in red, light blue and green, respectively.

(B) Immunosignals were detected at the expected molecular weight in lysates of HeLa cells transfected with Flag- and Myc-tagged human ZFTA and Flag-tagged mouse Zfta but not in the lysate of the mock transfected cells. Anti-GAPDH blot is shown as a loading control.

(C) Immunosignals of Flag- and Myc-tagged human ZFTA and Flag-tagged mouse Zfta using anti-Flag antibody were detected in the nuclei of transfected HeLa cells, suggesting that both are nuclear proteins. Immunosignals detected by the anti-human ZFTA antibody co-localised with those detected by anti-Flag antibody. Note that cells negative for anti-Flag staining were also negative for anti-human ZFTA staining (white arrows).

**Figure S2. Full scan images of western blot analysis shown in Fig. S1B**

Full scan images of anti-Flag (left), anti-human ZFTA (middle), and anti-GAPDH (right) immunoblots shown in Fig. S1B. "Shown" indicates the image shown in Figure S1B, while "replicate" is a sample for reproducibility and is not shown in Fig. S1B. Below the anti-Flag and anti-GAPDH blots, the signal-enhanced images are shown so that the edges of the membranes are visible.

**Movie 1. Ciliary beating of control E1 cells in 1/10 slow motion**

**Movie 2. Ciliary beating of *Zfta^tm1/tm1^* E1 cells in 1/10 slow motion**

**Movie 3. Live imaging movie showing the migration of fluorescent beads on the wholemount preparation of lateral wall of LV in control mouse.**

Anterior to the top, posterior to the bottom, dorsal to the right, ventral to the left.

**Movie 4. Live imaging movie showing the migration of fluorescent beads on the wholemount preparation of lateral wall of LV in *Zfta^tm1/tm1^* mouse.**

Anterior to the top, posterior to the bottom, dorsal to the right, ventral to the left.
